# Nutritional Intervention for the Intestinal Development and Health of Weaned Pigs

**DOI:** 10.3389/fvets.2019.00046

**Published:** 2019-02-21

**Authors:** Xia Xiong, Bie Tan, Minho Song, Peng Ji, Kwangwook Kim, Yulong Yin, Yanhong Liu

**Affiliations:** ^1^Laboratory of Animal Nutritional Physiology and Metabolic Process, Key Laboratory of Agro-Ecological Processes in Subtropical Region, National Engineering Laboratory for Pollution Control and Waste Utilization in Livestock and Poultry Production, Institute of Subtropical Agriculture, Chinese Academy of Sciences, Changsha, China; ^2^Department of Animal Science and Biotechnology, Chungnam National University, Daejeon, South Korea; ^3^Department of Nutrition, University of California, Davis, Davis, CA, United States; ^4^Department of Animal Science, University of California, Davis, Davis, CA, United States

**Keywords:** amino acids, feed additives, intestinal development, intestinal health, weaned piglets

## Abstract

Weaning imposes simultaneous stress, resulting in reduced feed intake, and growth rate, and increased morbidity and mortality of weaned pigs. Weaning impairs the intestinal integrity, disturbs digestive and absorptive capacity, and increases the intestinal oxidative stress, and susceptibility of diseases in piglets. The improvement of intestinal development and health is critically important for enhancing nutrient digestibility capacity and disease resistance of weaned pigs, therefore, increasing their survival rate at this most vulnerable stage, and overall productive performance during later stages. A healthy gut may include but not limited several important features: a healthy proliferation of intestinal epithelial cells, an integrated gut barrier function, a preferable or balanced gut microbiota, and a well-developed intestinal mucosa immunity. Burgeoning evidence suggested nutritional intervention are one of promising measures to enhance intestinal health of weaned pigs, although the exact protective mechanisms may vary and are still not completely understood. Previous research indicated that functional amino acids, such as arginine, cysteine, glutamine, or glutamate, may enhance intestinal mucosa immunity (i.e., increased sIgA secretion), reduce oxidative damage, stimulate proliferation of enterocytes, and enhance gut barrier function (i.e., enhanced expression of tight junction protein) of weaned pigs. A number of feed additives are marketed to assist in boosting intestinal immunity and regulating gut microbiota, therefore, reducing the negative impacts of weaning, and other environmental challenges on piglets. The promising results have been demonstrated in antimicrobial peptides, clays, direct-fed microbials, micro-minerals, milk components, oligosaccharides, organic acids, phytochemicals, and many other feed additives. This review summarizes our current understanding of nutritional intervention on intestinal health and development of weaned pigs and the importance of mechanistic studies focusing on this research area.

## Introduction

Weaning is the most challenging stage that has significant bearings on pig welfare and growth performance in swine industry. During weaning period, piglets are immediately imposed to a number of environmental and psychosocial stressors that predispose them to diarrhea and gut damage, which can adversely impact their survival at a very early and most vulnerable stage. The post-weaning mortality ratio is 6–10%, but sometime may rise up to 20%. Thus, in the last decade, animal nutritionists have made great effort to optimize feed formulation to meet requirement of newly weaned pigs, and to explore different nutritional factors or management that focus on promoting the overall health of weaned pigs. In addition, antibiotics used to be a powerful component in the herd health programs for protecting weaned pig health. It has been reported that global consumption of antibiotics in livestock production was estimated at 63,151 tons in 2010 and is projected to increase by 67% by 2030 (1). In the U.S., antibiotics use in livestock industry is estimated to account for 71% of the nation's annual antibiotic consumption (2). However, these practices also contribute to the spread of antibiotic-resistant pathogens in both livestock and humans, rising a significant public health threat. Use of in-feed antibiotics for production purpose in livestock industry is completely banned in the U.S. (3) starting in January 2017, which is remarkably increasing the challenge of keeping pigs healthy, especially in post-weaning period. Therefore, another urgent need in animal science society is to develop strategies to replace antibiotics for food-producing animals without hampering animal production. Although the manipulation of genetics, management, and health also plays substantially important role in protecting animal health and promoting their production performance, in the current review, we only focus on nutritional interventions on intestinal health of weaned pigs.

## Weaning Stress on Intestinal Development and Health

Many factors contribute to post-weaning stress, including hierarchy stress, new housing environment, transferring to solid feed, and others (4). Weaning stress is generally companying with reduced feed intake, poor growth performance, as well as increased disease susceptibility (5, 6). Weaning stress also negatively impacts intestinal development, physiology, microflora, and immunity as thoroughly discussed by other review articles (7–9). The focus of this review is to briefly highlight weaning stress on intestinal development and health by adding more recently published research.

### Weaning Stress on Pig Intestinal Physiology

Intestinal epithelium is characterized by rapidly proliferating cells in crypts, which then invaginating into the underlying mesenchyme and villi (10). The intestinal epithelial cells continuously and rapidly turn over in 4 to 5 days (11). The stem cells in crypts produce proliferating transit-amplifying cells that undergo a series of transitions, and ultimately differentiate into four differentiated cell types comprising one type of absorptive (enterocytes) and three types of secretory cell lineages (enteroendocrine cells, goblet cells, and paneth cells) (12). Absorptive enterocytes constitute up to 90% of epithelial cells in the crypt-villus axis (13). Paneth cells migrate to the base of crypts, whereas enteroendocrine cells and goblet cells migrate to villi (14). The proliferation, differentiation, and apoptosis of intestinal epithelial cells play important roles in intestinal development, maintenance, and recovery from tissue damage (7).

Several recently published research articles revealed the impacts of weaning stress on the expression of proteins and metabolites in enterocytes of piglets (15–20). Weaning significantly down-regulated the expression of proteins involved in the tricarboxylic acid cycle, β-oxidation, and the glycolysis pathway in the upper villus and middle villus of the jejunum in early-weaned pigs, but up-regulated proteins involved in glycolysis in crypt cells (15). During the post-weaning period, the expression of proteins related to various cellular metabolic or biological processes, such as energy metabolism, protein amino acid glycosylation, ion transport, mTOR signaling pathway, and differentiation and apoptosis, were reduced in jejunal differentiated epithelial cells (villus upper cells) of piglets (17). Proteins involved in the respiratory electron transport chain, Golgi vesicle transport, protein glycosylation, as well as the metabolism of nutrient such as lipids, monosaccharides, and nucleotides were also down-regulated in the jejunal differentiating epithelial cells (the middle villi cells) of piglets during the post-weaning period (20). These results indicated that weaning influenced energy metabolism, cellular macromolecule organization and localization, and protein metabolism, thereby further impacted the proliferation of intestinal epithelial cells in weaned piglets (18). In addition, polyamine metabolism and ornithine decarboxylase expression were also altered by weaning and may be used as a marker of intestinal growth and restitution in pigs (21).

Weaning stress could also induce tremendous morphological/physiological changes, such as villous atrophy and crypt hyperplasia (22, 23), which further disturb the digestive and absorptive capacity and performance of weaned pigs (4, 24). Brush border enzyme activities and electrolytes secretion in the small intestine have been used as important indicators of maturation and digestive capacity in weaned pigs (25, 26). Due to the change of diet, the activities of enzymes at brush border, such as lactase, sucrase, and maltase, are dramatically reduced between 3 and 5 days after weaning (27, 28). The malabsorption of nutrients in the small intestine is exacerbated by the reduced electrolytes absorption and secretion in newly weaned pigs (29).

The epithelial cells and the mucin layer in the small intestine provide the first line of defense to protect weaning pigs from various harmful microorganisms, toxins, or antigens in the intestinal tract (30). Gut permeability is straightly regulated by tight junction proteins, such as zona occludens 1, claudin, and occludin that are expressed by the epithelial cells (31). It has been reported that weaning stress reduced goblet cells number and mucin production, disrupted epithelial barrier function, increased intestinal permeability, lowered tight junction protein expression, and increased disease susceptibility in weaned pigs (32–34). It was observed that the intestinal barrier damage caused by weaning stress was not restored and returned to pre-weaning levels on d 7 post-weaning (35).

### Host-Microbial Nutrition Interactions in Post-weaning Gut Microflora Dysbiosis and Diarrhea

Porcine gut microbiome exhibits dynamic composition and diversity that shifts overtime (36, 37). The primary pig gut microbiota at birth was shaped by the sows' milk and featured with more abundance of lactic acid bacteria (38). However, weaning transition reduced the relative abundance of *Lactobacillus* group, increase *Clostridium* spp., *Prevotella* spp., *Proteobacteriaceae*, and *E. coli*, resulting in a loss of microbial diversity (39–41).

**Figure 1:**
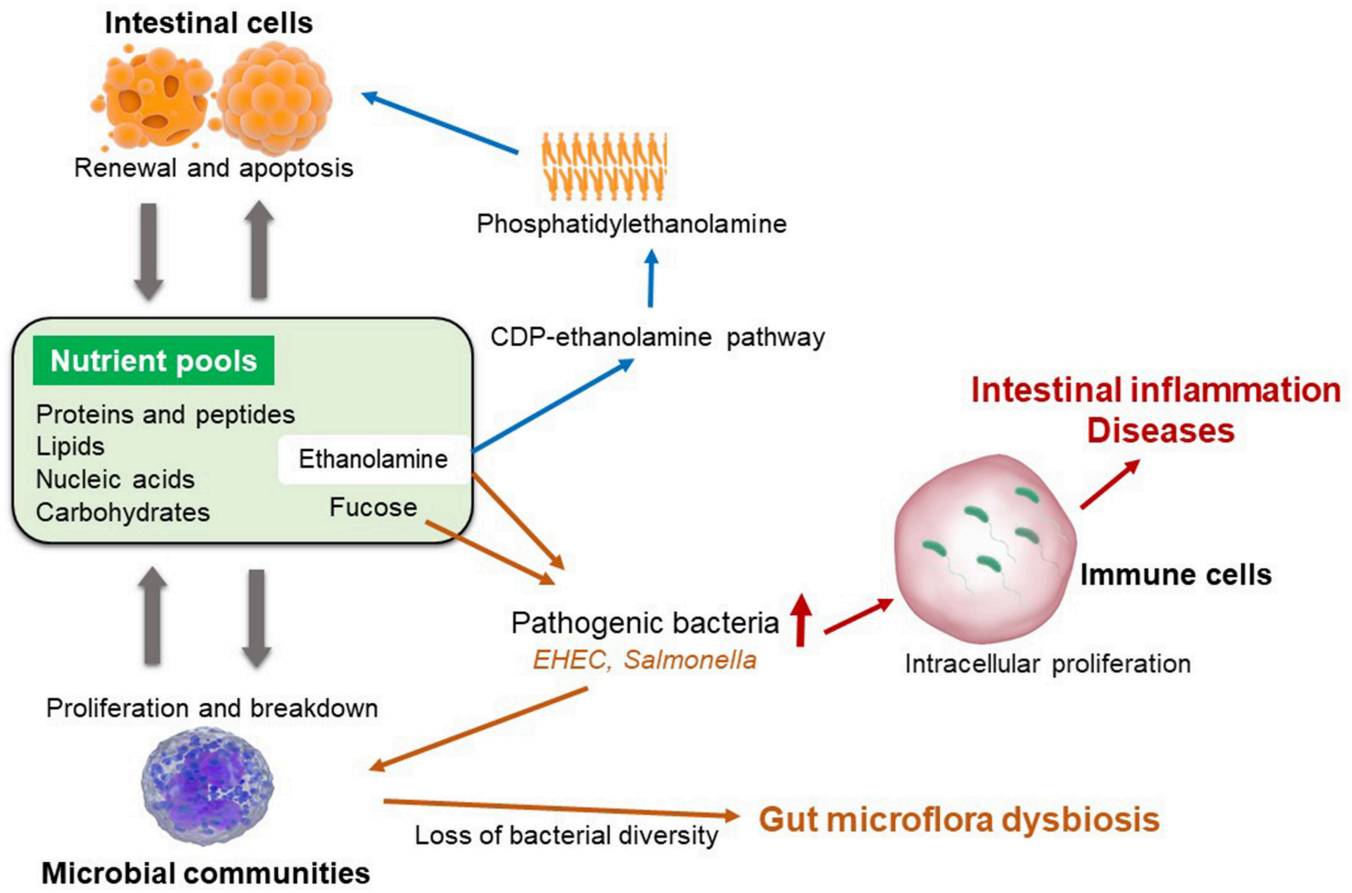
Maintenance of intestinal nutrient pool and the pathogenic baceterial specific nutrition metabolism.

The composition and diversity of gut microbiota of weaned piglets is also highly impacted by the levels and sources of dietary proteins or fibers that are offered to post-weaning pigs (42). Nutritional interactions between intestinal cells and gut microflora are remarkably important for the recycling and maintenance of gastrointestinal tract nutrient pool (Figure 1) (43–46). In contrast, a balanced nutrient pool is also critical for the renewal and proliferation of intestinal cells, as well as maintaining a balanced microbial community (12, 47). During the post-weaning period, piglets often have sharply reduced feed intake due to weaning stress. Hence, the nutrients for bacterial survival and proliferation is also limited. Pathogenic bacteria are able to utilize special nutrients (i.e., ethanolamine) that cannot be catabolized by commensal bacteria, thereby, enhance the expression of their virulence factors (48, 49). For instance, both *Salmonella* and enterohemorrhagic *E. coli* could use ethanolamine as carbon or nitrogen source to gain nutritional advantages in competing with other microflora (12, 48, 50). Enterohemorrhagic *E. coli* can also utilize fucose to activate type III secretion system, which facilitates the adhesion of those pathogenic bacteria to host enterocytes (46, 51). As a result, weaned piglets are more susceptible to intestinal inflammation and post-weaning diarrhea due to rapid proliferation of pathogenic bacteria and the loss of microbial diversity (52).

### Weaning Stress on Intestinal Mucosal Immunity

The barrier-related mucosal homeostasis is very important for the recognition of exogenous dangerous stimuli, but the same time it has to make sure our body is not hypersensitive to innocuous antigens (53). For example, in the intestine, epithelial cells are primarily responsible for fluid secretions and nutrients absorption, as well as providing a selective barrier against noxious antigens in the lumen. The cross-talk between intestinal epithelial cells and underlying lamina propria cells transfers immune-related signals to the local adaptive immunity, which subsequently help to maintain gut immune homeostasis (54).

The neonates are born with few lymphocytes and relatively low expression of co-stimulatory molecules (55, 56). In addition, the neonates also have a biased intestinal adaptive immunity due to a comparatively higher T helper 2 immune response rather than T helper 1 (57). To develop a stable number of lymphocytes in un-weaned pigs, it may take about 6 weeks (58). Therefore, newly weaned pigs at age of 2 to 4 weeks do not have mature intestinal immunity, which increase their disease susceptibility.

The impacts of weaning stress on intestinal immunity has been thoroughly revealed by McCracken et al. (59) and Pié et al. (60). Briefly, there are several major changes in intestinal immunity of weaned pigs compared with pre-weaning pigs. First, weaning sharply increases both intestinal CD4+ and CD8+ T lymphocytes in pigs on d 2 post-weaning (59) and enhances mRNA expression of inflammatory cytokines (e.g., TNF-α, IL-1β, IL-6, and IL-8) in the middle of jejunum during the first 2 day post-weaning (60). Those observations indicate that weaning induced a transient gut inflammation in pigs. Second, weaning stress up-regulates matrix metalloproteinase (i.e., stromelysin) by activating immune cells in the lamina propria, which may contribute to villus atrophy (59). Third, weaning stress may down-regulate the MHC I expression in jejunal mucosa of pigs, which is possibly due to the increased plasma cortisol concentration (59, 61). Fourth, the concentration of fecal IgA is continuously decreased from day 5 after birth and remained very low until at least 50 days of age, which may enhance the vulnerability of pre- and post-weaning piglets (62).

### Weaning Stress on Intestinal Oxidative Status

Weaning stress is also associated with increased oxidation processes, which leads to a high release of free radicals, also called reactive oxygen species [ROS; (63)]. The excessive production of ROS could modify certain cellular proteins and activate the up-regulation of pro-inflammatory cytokines, which may further negatively affect the expression of tight junction proteins and cause increased gut permeability (64, 65). Animal cells generally have complex and protective mechanisms to against the formation of oxidative stress, including prevention of ROS formation, ROS scavenging antioxidant systems, and elimination and/or reparation of damaged molecules (66). Therefore, the balance between oxidation and anti-oxidation is very important to cell integrity and health.

A series of antioxidant enzymes play critical roles to protect organisms against harmful pro-oxidants (67). For example, superoxide dismutase provides an efficient dismutation of ${\text{O}}_{2}^{-}$ into H_2_O_2_, which is scavenged by glutathione peroxidase and catalase (68). A study from Yin et al. (69) thoroughly investigated the impacts of weaning on the development of antioxidant system of pigs. They observed that plasma superoxide dismutase activity was decreased 1 day post-weaning and then gradually recovered at 3, 5, and 7 day post-weaning. They also observed that weaning down-regulated the expression of genes encoded superoxide dismutases (i.e., *CuZnSOD* and *MnSOD*) and glutathione peroxidases (i.e., *GPx1* and *GPx4*) in jejunum of piglets (69). A likely reason is that excessive ROS inhibits the phosphorylation and degradation of IκBs and Keap1, which, therefore, stimulates proteasomal degradation of Nrf2 and p65 and suppresses Nrf2 and p65 signals (69, 70).

## How to Define a Healthy Gut

A healthy gut is critically important to the overall metabolism, physiology, disease defense, and growth performance of weaned pigs. Recently, the item “gut health” has attracted much attention in the newly weaned pigs due to the negative effects of weaning stress. However, it still lacks a precise and unifying definition of “gut health.” Several review articles have comprehensively summarized timely information for this particular topic in newly weaned piglets (71–74) and provided slightly different definitions on “gut health.” Based on Kogut and Arsenault (71), a healthy gut was defined as the “absence/prevention/avoidance of disease so that the animal is able to perform its physiological functions in order to withstand exogenous and endogenous stressors.” Celi et al. (72) emphasized the importance of effective digestion and absorption of feed, effective structure and function of gut barrier, host interaction with gut microbiota, and effective immune status. The latest publication from Pluske et al. (74) stated that gut health should be more general and described as a generalized condition of homeostasis in the gastrointestinal tract. They remarked that the generalized criteria to assess gut health of weaned pigs could include effective nutrient digestion and absorption, effective waste excretion, a functional and protective gut barrier, a stable and appropriate microbial community, a functional and protective gut immunity, a minimal activation of stress/neural pathways, and the absence of diseases (74). It is not our intention to reiterate all details included in these publications and compare their definitions. In this regard, we completely agree that a healthy gut should enhance the overall capacity/ability of the host to respond and adapt to challenges/stress and should be concomitant with optimal performance as described by Pluske et al. (74).

## Nutritional Intervention on Intestinal Development and Health of Weaned Pigs

Many nutritional strategies have been applied to improve health and maximize the production of weaned pigs (75–78). Those strategies include but not limited to: optimization of feed formulation, utilization of low protein diet in post-weaning period, enhancement of feed processing and manufacturing, and supplementation of different feed additives. They are targeting different aims: (1) improvement of nutrient digestion and absorption, (2) regulation gut microbiota to more favorable bacterial species, and (3) immune modulation to enhance disease resistance of weaned pigs. In this review article, we will only focus on the impacts of several selected feed ingredients or additives (functional amino acids, phytochemicals, antimicrobial peptides, and short-chain fatty acids) on intestinal health of weaned pigs. Those feed additives may or may not have nutritional contribution to human or animal, but they play very important roles in health maintenance or regulation. Many other ingredients or additives are also shown promising results in weaned pig health, but will not be covered in the current article.

### Functional Amino Acids

A growing body of literature indicates that some of traditionally classified dispensable amino acids, such as, arginine, glutamine, glutamate, and proline play important roles in the regulation of gene expression, intracellular signaling pathways, nutrient metabolism, and oxidative defense (79–81). This group of amino acids is defined as functional amino acids (82). It has been known that the deficiency of a functional amino acid may impair the whole-body homeostasis. For example, dietary deficiency of arginine could result in metabolic, neurological, or reproductive dysfunction (83). The importance of functional amino acids has been thoroughly reviewed by Wu et al. (81) and Wu (84, 85). The major objective of this review section is to highlight recent published research articles focusing on the effects of functional amino acids on intestinal health and development of weaned pigs.

Arginine is remarkably deficient in sow milk (86, 87), but the concentration of arginine in tissue proteins in piglets are relatively higher compared with other amino acids (88). This observation has remarkably increased the research attention in the nutritional significance of arginine. It has been reported that supplementation of L-arginine (0.2 to 1%) enhanced growth performance and alleviated the negative effects of different insults or challenges in young pigs (81, 89–91). Supplementation of 0.4 to 0.8% L-arginine in pre-weaning diet enhanced intestinal growth and development in early post-weaning period (92). In addition, supplementation of 0.6% L-arginine enhanced small intestinal growth, goblet cell number in intestinal mucosa, intestinal heat shock protein-70 expression in weaned pigs (81). Increasing evidence confirmed the positive impacts of arginine on preventing intestinal dysfunction as a substrate for the synthesis of nitric oxide, polyamines, creatine, and protein (93). It was also reported that arginine could improve DNA synthesis and mitochondrial bioenergetics of intestinal epithelial cells, therefore improve the regeneration and repair of the small intestinal mucosa in animals (94). The underlying biochemical mechanisms may be closely related to the activation of PI3k-Akt pathway, mTOR and TLR4 signaling pathways, and/or the enhanced intracellular protein turnover (94, 95). Moreover, the increased nitric oxide from arginine metabolism could also regulate intestinal blood flow, integrity, secretion, and epithelial cell migration (96).

Besides arginine, other functional amino acids in the arginine family, have been also well investigated in the last decades, including glutamine, glutamate, aspartate, proline, etc. For example, it was reported that the administration of proline improved mucosal proliferation, intestinal morphology, as well as tight junction and potassium channel protein expression in early-weaned piglets (97). Dietary supplementation of glutamine was also shown to prevent intestinal atrophy, increase enzyme activities, and promote growth performance of weaned pigs (98). One dipeptide that is composed of glutamine (glycyl-glutamine), appear a great substitute for glutamine to increase intestinal integrity and enzyme activities and growth performance of weaned pigs (99–101). Another dipeptide, alanyl-glutamine, also has the biological effects similar to free glutamine, as regarding their effects on proliferation, mitochondrial respiration, and protein turnover in the porcine intestinal cells (102). Alanyl-glutamine may be another effective substitute for glutamine as energy and protein sources in the intestinal tract, which has to be further investigated with *in vivo* animal model. Several mechanisms are highly involved in the benefits of glutamine or glutamine dipeptides on intestinal health. First, glutamine, glutamate, and aspartate could provide major fuel for small intestinal epithelial cell proliferation and provide energy required for intestinal ATP-dependent metabolic processes (103). Second, catabolism of glutamine provides precursors for polyamine synthesis, which is important for proliferation, differentiation, and repair of intestinal epithelial cells (104). Third, glutamine is also a major precursor for the synthesis of glutathione, an important antioxidant in cells regulating the homeostasis of free radicals (105, 106). Fourth, glutamine supplementation may enhance intestinal secretory IgA production via regulating the intestinal microbiota and/or T cell-dependent and T cell-independent pathways (107).

Although it is beyond the scope of functional amino acids, several indispensable amino acids, such as tryptophan and sulfur amino acids, have also attracted large attention recently (108–110). A growing evidence has revealed that supplementation of these amino acids beyond the current NRC requirement brought positive effects on intestinal health of weaned pigs by regulating host physiology, metabolism, oxidative status, and immunity (108–110). The modification of gut microbiota and their metabolites by these amino acids was also highly correlated to the enhanced gut barrier functions of weaned pigs (109).

### Phytochemicals

Phytochemicals, naturally occurring plant chemicals/metabolites, are one of most powerful candidates as potential alternatives to in-feed antibiotics because of various biological functions. First, most of phytochemicals exhibit a wide spectrum of antibacterial activities against both gram-negative and gram-positive bacteria, including *E. coli, Salmonella*, Clostridium, Mycobacterium, etc. (111, 112). Second, certain phytochemicals have been recognized as potential anti-viral agents (113, 114), which is probably beyond provision of antibiotics. Third, the immune-regulatory activities of certain phytochemicals have been identified in both human and animal models (114–118). Last but not the least, phytochemicals could act as antioxidants to remove free radicals from the body and protect animals from oxidative damage (119). Several commonly used phytochemicals and their main components are summarized in Table 1.

**Table 1:** Several commonly used phytochemicals and their main components exhibiting different biological activities, modified from Liu (120).

**Scientific name**	**Common name**	**Main components**	**Biological activities**
*Allium saticum*	Garlic	Allicin	Antimicrobial Anti-inflammatory
*Capsicum*	Pepper	Capsaicin	Antimicrobial Anti-inflammatory
*Cinnamomum verum* J. Presl *Cinnamomum osmophloeum*	Cinnamon	Cinnamaldehyde	Antimicrobial Anti-inflammatory Antioxidant
*Eugenia caryophyllus* Spreng. *Eugenia caryophylata* Thunb	Clove	Eugenol	Antioxidant
*Foeniculum vulgare*	Fennel	Anethol	Antioxidant
*Funicular vulgare*	Fennel	Anethol Eugenol	Antimicrobial
*Origanum vulgare* spp. *Origanum onites* *Origanum minutiflorum*	Oregano Thyme	Carvacrol	Antimicrobial Anti-inflammatory Antioxidant
*Punica granatum*	Pomegranate	Ellagic acid	Anti-inflammatory
*Syzygium aromaticum* (L.) *Eugenia caryophyllata*	Cloves Fennel	Anethol Eugenol	Antimicrobial Anti-inflammatory
*Thymus vulgaris* L. *Thymbra spicata*	Thyme Fennel	Thymol Carvacrol Terpinene	Antimicrobial Anti-inflammatory Antioxidant
*Zanthoxylum schinifolium*	Rutaceae	Citronellal β-Phellandrene	Anti-inflammatory
*Zingiber officinale*	Ginger	Curcumin Gingerol	Antimicrobial Anti-inflammatory Antioxidant

The protective effects of phytochemicals on poultry and livestock have been thoroughly reviewed in Lillehoj et al. (121). Previous research revealed that dietary supplementation of phytochemicals enhanced disease resistance (i.e., reduced frequency of diarrhea) and growth performance (114, 122, 123). These benefits were likely driven by improved gut health, such as, improved intestinal barrier integrity (122, 123). For example, supplementation with phytochemicals extracted from different seasonings improved intestinal villi height and upregulated mRNA expression of the *MUC2* gene in ileum (118). Feeding capsicum oleoresin from pepper, turmeric oleoresin or curcumin extracted from ginger up-regulated the expression of genes related to tight junction (e.g., genes encode claudins and occludin) and cell-cell junctions in the ileum of *E. coli* challenged pigs (118, 124). A recent publication from Yuan et al. (125) also reported that the flavones extracted from the leaves of *Eucommia ulmoides* enhanced intestinal morphology and integrity of diquat challenged pigs by improved intestinal barrier function.

The immuno-regulatory and antioxidant properties of phytochemicals are also responsible for their positive effects on animal health. Lang et al. (126) reported that garlic extract could inhibit the secretion of chemokines from intestinal epithelial cells, thus suppress the recruitment of various circulating leukocytes into the inflamed tissue. Dietary supplementation of phytochemicals (10 mg/kg of capsicum oleoresin, garlic, or turmeric oleoresin) downregulated the expression of genes related to antigen processing and presentation and other immune response-related pathways, indicating that these phytochemicals may attenuate the immune responses caused by *E. coli* infection (118). Supplementation of flavones extracted from the leaves of *Eucommia ulmoides* also alleviated the inflammatory responses of weaned pigs induced by diquat (125). Several commonly used phytochemicals (extracts from oregano, thyme, ginger, fennel, pepper, clove, basil, cinnamon, garlic, mint etc.) are also showing strong antioxidant activities in both *in vitro* cell culture and *in vivo* animal models (127–130). The antioxidant property of phytochemicals is mainly associated with the phenolic compounds that have high reactivity with peroxyl radicals, which are free radical species for the oxidation of proteins and lipids (131, 132). Otherwise, surfur-containing volatiles in garlic extracts express strong antioxidant activity due to the formation of unstable degradation products as radicals-trapping agents (129). However, limited research have been reported the effects of phytochemicals on intestinal oxidative status/responses of weaned pigs.

### Antimicrobial Peptides

Antimicrobial peptides, also known as host defense peptides, have been considered as potential alternatives to antibiotics in livestock and poultry (133–135). Antimicrobial peptides are polypeptides, naturally produced by different organisms from prokaryotes to mammals. Therefore, antimicrobial peptides could be directly isolated from bacteria, insects, plants, and vertebrates, or could be synthesized as recombinant molecules (136). They are small and positively charged, and contain both hydrophobic and hydrophilic regions. The majority of antimicrobial peptides are belonged to either defensins or cathelicidin family, whereas defensins are further divided into α-, β-, θ-defensins on the basis of the spacing patterns of their cysteine residues (134). Compared with cathelicidins that are highly expressed in mammalian neutrophils, defensins are more abundant in epithelial and phagocytic cells in different tissues, including intestinal mucosa (137).

Antimicrobial peptides possess a strong and large-spectrum activity against gram-negative and gram-positive bacteria, fungi, parasites, and viruses (138). Compared with traditional antibiotics, one obvious advantage of antimicrobial peptides is they could kill pathogenic bacteria (e.g., *P. aeruginosa* and *Staphylococcus aureus*) that are resistant to specific antibiotics (134, 139). As mentioned above, most antimicrobial peptides are small, positively charged, and amphipathic molecules that allow them to actively interact with bacterial membranes through different models (barrel-stave model, carpet model, or toroidal-pore model) (140, 141). As a consequence, antimicrobial peptides could disturb the structure of cell membrane, penetrate into cells, regulate intracellular pathways, and/or cause cell death. Other mechanisms may be also involved in the antibacterial properties of antimicrobial peptides, such as inhibiting cell wall synthesis, suppressing protein and nucleic acid synthesis, and inhibiting enzymatic activities in bacteria (142).

The protective effects of antimicrobial peptides on intestinal health have been reported in weaned pigs. Supplementation of recombinant lactoferrin increased gut morphology (e.g., greater villi height) and growth performance of piglets (143). Xiao et al. (144) reported that feeding 0.4% of a mixture of antimicrobial peptides (including bovine lactoferrin and plant defensins) and active yeast alleviated the negative effects of mycotoxin by increasing intestinal integrity and reducing intestinal permeability of weaned pigs. Several defensins were shown to enhance mucosa barrier function by up-regulating the expression of mucin and tight junction proteins (145). The potential benefits of antimicrobial peptides are also related to other modes of action, such as regulating immune responses and gut microbiota (146). Supplementation of recombinant lactoferrin or lactoferramoin-lactoferricin increased *Lactobacillus* and *Bifidobacterium* counts but reduced total *E. coli* and *Salmonella* in the small intestine of weaned pigs (146, 147). Addition of cecropin A/D reduced incidence of diarrhea and enhanced intestinal *Lactobacilli* counts in *E. coli* challenged piglets (148). As reviewed in Zasloff (136), fully processed active peptides probably act as epithelial “preservatives” to protect host against intestinal infectious agents. They may also work as effector molecules of innate and adaptive immunity by regulating inflammatory responses and chemotactic activity in pigs (149, 150).

There are two ways to incorporate the benefits of antimicrobial peptides into animal health and nutrition. One is direct supplementation of exogenous antimicrobial peptides to animal feed, while the other one is to use dietary supplements/ingredients to stimulate the secretion of endogenous antimicrobial peptides by the host (135). Although exogenous or recombinant antimicrobial peptides have shown a great potential to be used as alternatives to replace antibiotics, the effectiveness of those candidates should be carefully verified because the majority of exogenous antimicrobial peptides would be digested in the upper gastrointestinal tract without reaching to the lower part where most pathogens reside. Therefore, the stimulation of endogenous antimicrobial peptides secretion by nutritional manipulation may be a better approach. For instance, Robinson et al. (135) have completely reviewed the regulation of antimicrobial peptides synthesis by butyrate and vitamin D in livestock and poultry and pointed out the importance of antimicrobial peptides-inducing compounds in antibiotic-free animal production.

### Short-Chain Fatty Acids

Short-chain fatty acids (SCFAs) are fatty acids with a chain of <6 carbon atoms, which are primarily produced by hindgut fermentation of dietary fiber. The SCFAs are a major fuel source for colonocytes, and are essential for maintaining the normal metabolism of colon mucosa, including colonocyte growth and proliferation (151, 152). In particular, as much as 90% of butyric acid is metabolized by colonocytes (153). However, the benefits of SCFAs is probably not limited to the colon: (1) SCFAs may function as a direct energy source for enterocytes, thus, increase proliferation and reduce apoptosis of enterocytes (154, 155), (2) SCFAs may modulate the expression of genes involved in gut motility, host defense, and inflammatory responses (154, 156), (3) SCFA could stimulate the formation of intestinal barrier and protect intestinal barrier disruption (157), and (4) SCFA may affect the composition of gut microbiota (158–160). The most abundant SCFAs in the gastrointestinal tract are acetate, propionate, and butyric acid. Despite being the least abundant of the 3 primary SCFAs, butyric acid has attracted significant research attention due to its' importance of maintaining gut health in both human and animals.

Butyric acid, also known as butanoic acid, is one of the SCFAs that are produced by microbial fermentation in the gastrointestinal tract of pigs (161). Especially, the propionic and butyric acids produced in the gastrointestinal tract are considered important metabolites that have antibacterial effects on pathogenic bacteria (162). In particular, butyrate has received particular attention and has been widely investigated as an attractive potential alternative to replace in-feed antibiotics. Addition of butyric acid directly to a swine diet may be limited because of its highly volatile and corrosive characteristics (163). Therefore, some products of butyric acid have been used in combined forms with calcium or sodium.

It has been reported that dietary supplementation of 0.1% sodium butyrate reduced diarrhea, enhanced gut integrity, increased serum IgG, but decreased serum pro-inflammatory cytokines in weaned pigs under normal conditions (158, 159, 164, 165). Machinsky et al. (166) also observed a positive effect of sodium butyrate on the protein digestibility of pigs. Another alternative form of butyrate is glyceryl tributyrate, also called tributyrin. Tributyrin is a naturally present triglyceride in butter at the minute amounts. The major advantage of tributyrin vs. sodium butyrate is that tributyrin is a delayed release source of butyrate. Tributyrin stays intact in the stomach and is slowly released as butyrate and/or monobutyrin in the small intestine where pancreatic lipase appears. Feeding 0.1% tributyrin reduced intestinal injury caused by intrarectal administration of acetic acid, as indicated by improved tight-junction formation and activated epidermal growth factor receptor signaling (167). Supplementation of tributyrin also improved the growth and intestinal barrier functions in intrauterine growth-restricted piglets (168).

**Figure 2:**
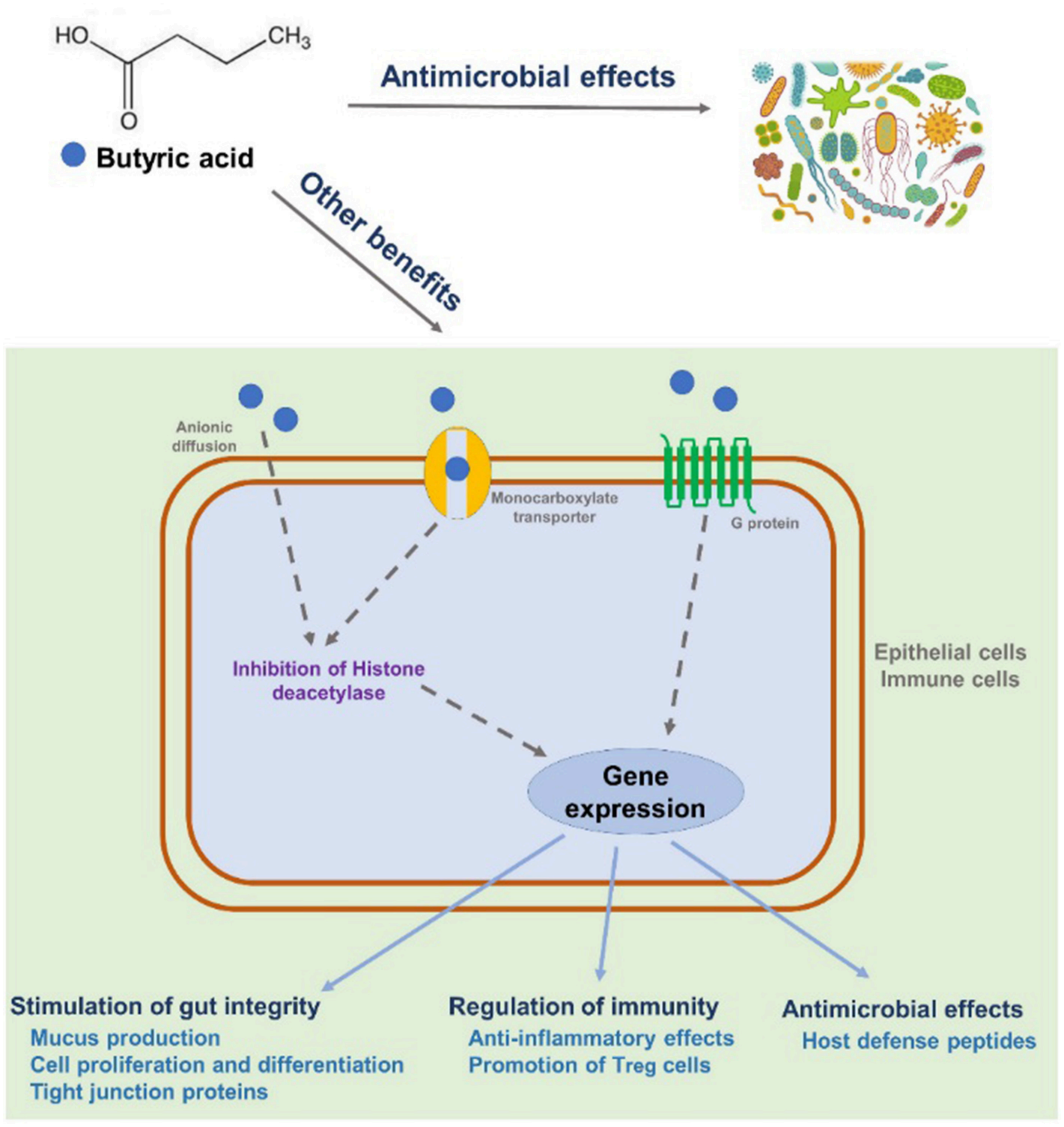
Proposed mechanisms of action on the beneficial effects of butyric acid.

Despite many years of research, the exact mechanism of action of dietary butyrate supplements has not been fully elucidated, but the following mechanisms have been proposed (Figure 2). Butyric acid penetrates into epithelial cells either by simple diffusion or monocarboxylate transoporter (169). Butyric acid could also bind to G-protein-coupled receptor expressed in epithelial cells or immune cells. The binding will mediate a cascade of immune regulation (170). A brief summary for the anti-microbial and/or immuno-regualtory effects of butyric acid is shown below. First, butyric acid regulates a large amount of gene expression as one of histone deacetylase inhibitors by removing acetyl groups from the N-terminal tail of the histones (171, 172). Recent studies also revealed that the inhibition of histone deacetylase is highly correlated to the regulation of inflammatory responses and immunity by butyric acid in both human and rodents (157, 173, 174). Second, butyric acid and its derivatives have been shown to possess strong antimicrobial activity against both gram-positive and gram-negative pathogenic bacteria both *in vivo* and *in vitro* (175, 176). The antimicrobial activity of butyric acid is likely due to the ability of this acid to penetrate the bacterial cell wall and acidify the cell cytoplasm, thereby causing bacterial death (177). Third, butyric acid could enhance the expression of host defense peptides in different types of porcine cells, which is remarkably important in modulating host immune system and against a range of pathogens including antibiotic-resistant strains (178, 179). Last but not least, butyric acid may be able to alleviate intestinal injury by promoting tight-junction formation (167, 180).

## Conclusions

A healthy gut is extremely important, as the gut is a nutrient digestion and absorption organ, a chemo-/nutritional sensing organ, as well as the largest immune organ in the body. The young pigs in post-weaning period have limited luminal nutrition supply and are immediately imposed to tremendous challenges, which cause changes in the structure and function of the intestinal tract. These changes may include but not limited to disrupted intestinal structure, reduced digestive and absorptive capacity, damaged intestinal barrier, loss of microbial diversity, and unbalanced intestinal immune homeostasis. A large amount of research have been conducted to increase our understanding of the importance of gut health on animal production and performance, although the definition of a healthy gut is still not unified. Currently, the most summarized and generalized one is that a healthy gut may contain several key criteria, such as, effective nutrient digestion and absorption, effective waste excretion, a functional and protective gut barrier, a stable and appropriate microbial community, a functional and protective gut immunity, a minimal activation of stress/neural pathways, and the absence of diseases. To promote gut health of weaned pigs, particularly under the restriction of the use of antibiotics in feed, a wide arrange of nutritional interventions have been proposed and investigated. Increasing evidences show that supplementation of extra functional amino acids or specific phytochemicals could provide very positive impacts on intestinal integrity and immunity of weaned pigs. Antimicrobial peptides and their inducing compounds such as butyrate derivatives have also emerged as a potentially viable alternative to replace antibiotics and to maintain intestinal health. There are much more candidates of feed additives/nutritional interventions than the four listed in this review, which may be effective in regulating intestinal environments and enhancing weaned pig performance. It is very important to keep in mind that the efficiencies of each candidate may differ on the basis of their modes of action, the basal diet formulation, and the health status of pigs. Moreover, the importance of omics approaches (i.e., metagenomics, transcriptomics, proteomics, metabolomics, etc.) should be highly recognized as well, although it is not discussed in the current review. These novel approaches have been widely adopted to explore the mechanisms of nutritional interventions on animal health and production by investigating the impacts of nutrition on intestinal microbiota and their metabolites, and the interactions of nutrition, genes and their encoded products (proteins and peptides, etc.).

## Author Contributions

All authors listed have made a substantial, direct and intellectual contribution to the work, and approved it for publication.

### Conflict of Interest Statement

The authors declare that the research was conducted in the absence of any commercial or financial relationships that could be construed as a potential conflict of interest.
